# Essential meiotic structure-specific endonuclease1 (*EME1*) promotes malignant features in gastric cancer cells via the Akt/GSK3B/CCND1 pathway

**DOI:** 10.1080/21655979.2021.1999371

**Published:** 2021-12-11

**Authors:** Zhiguo Guo, Erbo Liang, Wei Li, Leilei Jiang, Fachao Zhi

**Affiliations:** a Guangdong Provincial Key Laboratory of Gastroenterology, Institute of Gastroenterology of Guangdong Province, Department of Gastroenterology, Nanfang Hospital, Southern Medical University, Guangzhou, Guangdong, China; b Department of Endocrinology, Suzhou Hospital of Anhui Medical University, Suzhou, Anhui, China; c Department of Gastroenterology, Suzhou Hospital of Anhui Medical University, Suzhou, Anhui, China

**Keywords:** *EME1*, gastric cancer, Akt/GSK3B/CCND1, proliferation, invasion

## Abstract

DNA damage plays a key role in various biological processes involved in malignant disease, the role of the DNA damage repair gene *EME1* (essential meiotic structure-specific endonuclease 1) in gastric cancer (GC) development is unknown. This work aimed to investigate expression and role of *EME1* in tumorigenesis. Quantitative real-time polymerase chain reaction (qRT-PCR), immunoblot, cell viability and dual-luciferase reporter assays, RNAi and gene transfection, and immunofluorescent staining were performed to assess *EME1* regulation in GC tumorigenesis. Further, mouse xenografts were established for *in vivo* mechanistic studies. *EME1* was found to be upregulated in both gastric cancer cells and clinically obtained tumors. Additionally, *EME1* levels were strongly associated with the differentiation level of GC and lymph node metastasis. *In vivo* and *in vitro* knockdown of *EME1* markedly suppressed the proliferative, migratory, and invasive abilities of GC cells and enhanced apoptotic cell death and cell cycle arrest rates. Mechanistically, *EME1* modulated Akt/GSK3B/CCND1 signaling. *MYB* may also have contributed to *EME1*-dependent gastric carcinogenesis. Elevated *EME1* expressions may enhance the proliferative and metastatic abilities of GC cells, thereby acting as a tumor-promoting factor via Akt. These findings reveal that *EME1* is an important biomarker for GC prognosis and treatment in humans.

**Abbreviations**: Essential meiotic structure-specific endonuclease 1 (*EME1*); MYB proto-oncogene (*MYB*); Cell counting kit-8 (CCK-8); 4,6-diamimo-2-phenyl indole (DAPI); Quantitative real-time PCR (qRT-PCR); Gastric cancer (GC); Immunofluorescence (IF); Small interfering RNA (siRNA); Small hairpin RNA (shRNA); Alpha serine threonine-protein kinase (Akt); Glycogen synthase kinase 3 beta (GSK3B); Cyclin D1 (CCND1); Glyceraldehyde-3-phosphate dehydrogenase (GAPDH); Disease-free survival (DFS); Overall survival (OS); Negative controls (NC); American Joint Committee on Cancer (AJCC); Coding sequence (CDS); Lymph node metastasis (LNM); Tris-Buffered Saline-Tween-20 (TBST); Horseradish Peroxidase (HRP); Electrochemiluminescence (ECL); Polyvinylidene Fluoride (PVDF); Excision repair cross complementation group 1 (ERCC1).

## Introduction

Recent statistics have shown that gastric cancer (GC) is prevalent in high-income regions of Asia-Pacific and East Asia. GC represents the fifth deadliest malignancy worldwide [1]. In 2020, GC comprised 5.6% of all incident cancers and 7.7% of global cancer-related deaths [2]. As patients with early stage GC show no symptoms, most of them are diagnosed at an advanced or metastatic stage, which leads to a poor prognosis. GC occurrence is a complex process involving a *Helicobacter pylori* infection, genetic susceptibility of the host, and additional environmental factors. It is, therefore, critical to comprehensively understand the pathogenic mechanisms of gastric carcinogenesis and thereby improve patient prognosis. In addition, it is imperative to identify additional biomarkers for treating GC.

Oxaliplatin has superior efficacy compared to docetaxel in GC patients with a low excision repair cross complementation group 1 (ERCC1) mRNA expression [3]. Proteogenomic analysis demonstrating the correlation between mRNA and protein abundance reveals potential oncogenic and tumor-suppressive molecules associated with GC patient survival [4]. In addition, DNA replication stress and chromosomal instability induced by oncogenes are considered as drivers of tumorigenesis, and are associated with drug resistance and poor prognosis. Upon cell exposure to replication stress, the endonuclease MUS81-EME1, with structural specificity, induces the generation of chromosomal gaps/breaks at common fragile sites (CFSs), along with a cleavage of under-replicated DNA involved in mitotic DNA synthesis. This results in defective chromosomal segregation and facilitates cancer development [5,6].

Essential meiotic structure-specific endonuclease 1 (*EME1*) substantially contributes to repairing double-strand breaks, mediating aberrant Holliday junctions and replication fork structures, and maintaining genomic stability. Recent studies also indicate role of *EME1* in cell volume regulation, proliferation, and migration. These processes are associated with bladder cancer recurrence [7], especially in glioma and prostate cancer cells [8,9]. An association between *EME1* upregulation and reduced survival has been demonstrated in cases of pancreatic cancer [10]. MacGregor et al. [11] examined the mRNA levels of 280 DNA repair (DNAR) genes prior to treatment of esophageal adenocarcinoma patients, followed by regression analyses of surgery, disease-free survival (DFS), and overall survival (OS). Their results showed that *EME1* is associated with reduced OS, indicating that *EME1* is a prognostic biomarker in esophageal adenocarcinoma. Thus, *EME1* may play a key role in tumorigenesis; however, it is unclear whether *EME1* expression in GC patients is associated with gastric carcinogenesis.

The purpose of this study was to explore the role of *EME1* in the proliferation, apoptosis, and metastasis of GC cells. The relationship between *EME1*, Akt, and *MYB* in the regulation of gastric cancer was also studied. Our results reveal the potential role of *EME1* in GC development and its molecular mechanism in tumor progression while suggesting that *EME1* may be a potential therapeutic target for GC.

## Materials and methods

### Patients and specimens

GC and adjacent tissue samples were obtained from the surgical specimen archives of the Suzhou Hospital of Anhui Medical University. The study was approved by the Biomedical Ethics Committee of Anhui Medical University (20,200,663). All patients provided signed informed consent. This study followed the International Ethical Guidelines for Biomedical Research Involving Human Subjects (CIOMS). None of the patients received preoperative radiation therapy or chemotherapy. Disease staging utilized the American Joint Committee on Cancer (AJCC) 8th edition GC staging criteria [12], and 36 pairs of primary GC and adjacent noncancerous tissue specimens were collected.

### Cell culture

Human gastric epithelial cells, GC, AGS, and MGC-803 cells (ATCC) were used in this study. Dulbecco’s Modified Eagle Medium (DMEM; Gibco, Thermo Scientific, Shanghai, China) containing 10% fetal bovine serum (FBS) (Merck, Shanghai, China), 100 U/mL penicillin, 100 µg/mL streptomycin (Thermo Scientific, Shanghai, China) and 8 µg/mL Tylosin tartrate (antimicrobial agent inhibiting mycoplasma; Sigma, USA) were used for the cell culture.

### Plasmid construction and cell transfection

The cDNA sequence of full-length human *EME1* was subjected to PCR for *in vitro* amplification. The recombinant plasmid was constructed by cleaving the PCDNA3.1(+) vector (Invitrogen) using the restriction endonucleases KPNI and XBAI and ligating the *EME1* fragment to the vector, using T4 DNA ligase. The successful construction of the recombinant plasmid was validated via DNA sequencing. Cell transfection was performed using siRNA (Ribobio, China) targeting *EME1* to knockdown *EME1*. Lipofectamine 2000 (Invitrogen) was used for transfection in manner as recommended by the manufacturer. For animal experiments, lentiviruses with the *EME1* overexpression and NC were provided by GenePharma Biotech to transfect AGS and MGC-803 cells and establish cell lines stably silenced for *EME1*. For transfection, the cell confluence was 60%–70%. Lentiviruses and plasmids were labeled with green fluorescent protein (GFP) and transfection were detected using a fluorescence microscope. The ratio of the number of green fluorescent cells to the total number of cells was counted using ImageJ software to calculate the transfection efficiency. The calculation results indicated a transfection efficiency of >80% (Supplementary Figure 2a, b). DNA plasmids were isolated from *Escherichia coli* extracts containing methylesterase to block methylation-restricted endonucleases.

### Xenograft assays

Animal experiments were approved by the Experimental Animal Ethics Committee of Anhui Medical University (2,020,587). Specific-pathogen-free (SPF) BALB/C nude mice (4 weeks old) were used for the experiments. Food and water were provided ad libitum. Flow cytometry-sorted primary GC cells were collected, and 2 × 10^6^ shEME1-transferred AGS cells with luciferase markers in 150 µL phosphate-buffered saline (PBS) were injected into the lateral abdomen of mice (Supplementary Figure 3a, b). Bioluminescence signals in mice were observed *in vivo* using an IVIS100 imaging system (5 per group, NC group vs. siEME1 group). All mice were euthanized by 2% pentobarbital overdose on day 56, and their tumor volumes and weights were measured. Tumor specimens obtained at the indicated time points were paraffin-embedded and stained with hematoxylin and eosin (H&E) stain.

**Small interfering RNAs (siRNAs**), small hairpin RNA (shRNA), **GapmeRs, and transfection**

siRNA synthesis was performed by GenePharma (Shanghai, China). Qiagen (USA) provided antisense LNA™ GapmeRs for all four transcript variants of EME1 and NC. siRNA and GapmeR transfections were performed using Lipofectamine 2000 (Life Technologies, USA) at 20 nM and 10 nM, respectively, according to the manufacturer’s instructions. After 48 h, cell collection was performed using various assays. The shRNA clones were then processed, as described ahead. First, a single-stranded DNA oligomer with an interference sequence was synthesized, cooled to room temperature in a water bath for 15 min to produce double strands, and was then directly ligated to the pLenRGPH lentivirus vector using T4 DNA ligase through the EcoRI and BamHI cleavage sites contained at both ends. The ligation product was then transferred into the prepared bacterial competent cell DH5α. Next, the positive recombinant was identified via PCR and subsequently sent for sequence verification. The clones confirmed by successful sequencing formed the shEME1 lentivirus vector. All siRNA and shRNA sequences used in this study are listed in Supplementary Table 1.

### CCK-8 and colony formation assays

CCK-8 (Biolite, USA) was used to assess cell viability and determine the effect of *EME1* on cell proliferation. A total of 2 × 10^3^ cells (control or treated) per well were inoculated into 96-well plates, and 10 μL CCK-8 solution was added to each well starting the day following cell plating. The setup was incubated at 37°C for 2 h. Next, the absorbance at 450 nm was measured on a microplate reader. A clonogenic assay was performed by seeding 900 cells in 6-well plates and incubating them for 14 days. After fixation with ethanol for 30 min, the cells were stained with 0.1% crystal violet for 20 min.

### Assessment of cell cycle distribution and apoptosis

Cells, after reaching 80% confluency, were washed twice with chilled PBS. To assess the cell cycle distribution, cell fixation was performed overnight at 4°C with pre-chilled 70% ethanol, followed by two chilled PBS washes and filtration with a 0.05 mm cell strainer. Next, the specimens were incubated with 50 μg/mL propidium iodide (PI), 100 μg/mL RNase A, and 0.2% Triton X-100 in PBS at 4°C for 30 min. Cellular DNA content was analyzed via flow cytometry on a C6 Plus flow cytometer (BD Biosciences, USA). To assess apoptosis, cells treated with EDTA-free trypsin were collected and stained with the PE Annexin V Apoptosis Detection Kit (BD Pharmingen) for 20 min at room temperature, as directed by the manufacturer.

### Transwell invasion and scratch assays

In the Transwell invasion assay, Transwell chambers were placed in 24-well plates. Next, 500 μL of serum-free RPMI1640 with 10% FBS was added to the bottom compartments, and cells were inoculated in 200 μL of serum-free RPMI1640 in upper chambers. After 24–48 h, cell fixation was performed using methanol, followed by staining with crystal violet. Cell morphology was observed under a microscope. In the scratch assay, 1.5 × 10^5^ cells were inoculated, transfected, and cultured in 12-well plates. After adhesion to the bottom of the well, fine scratches of uniform width were made along the center of each well with a 100 μL pipette tip. Image acquisition was performed with an inverted microscope. The 12-well plate was marked, and the same field of view was positioned again. The samples were incubated at 37°C for 24 h, and the cells were rinsed thrice with PBS to remove surrounding cell debris.

### Quantitative real-time PCR (qRT-PCR)

Total RNA was extracted using TRIzol (Life Technologies). Retrotranscriptional cDNA was acquired from GenStar, A214-10, Beijing, China. The RT enzyme was inactivated by placing the sample in a water bath at 42°C for 1 h, and then at 70°C for 10 min. The cDNA of the reverse transcription product obtained was used as a template in a SYBR Green Real-time PCR Master Mix (Invitrogen, Carlsbad, CA, USA). M-MLV reverse transcriptase (Promega) was used in the reverse transcription, and qRT-PCR was performed using an Agilent Mx3000P qPCR system (Agilent Technologies Inc, California, USA) with specific primers. The total primers are shown in Supplementary Table 2.

### Western blotting

Tissues or cells were washed with cold PBS and homogenized. A RIPA cracking solution was added to the solution at 4°C for 1 h. The protein concentration was determined using the BCA method, with cellular protein added to a 96-well plate and incubated with the BCA working solution. The absorbance at 562 nm was then measured using a microplate reader. SDS-polyacrylamide gels were prepared for Western blotting. Cellular proteins were denatured. Samples were centrifuged at 13,000 rpm for 15 minutes at 4°C, and then electrophoresed at a constant pressure in loading buffer. Following electrophoresis, protein bands were transferred onto Polyvinylidene Fluoride (PVDF) membranes, which were stained with Ponceau S solution (Absin Bioscience Inc, Shanghai, China) and blocked with skimmed milk. The membranes were separately incubated overnight at 4°C with GAPDH (Abcam ab181602), EME1 (Abcam ab88878), P-Akt (CST 4060), Akt (CST 4691), p-GSK3B (Ser9) (CST 5558), GSK3B (CST 12456), P-CCND1 (Abcam ab62151), and CCND1 (Abcam ab134175) antibodies rinsed with Tris-Buffered Saline-Tween-20 (TBST). Then, the membranes were further incubated with Horseradish Peroxidase (HRP)-labeled secondary antibodies. Finally, an electrochemiluminescence (ECL) was used for visualization.

### Immunohistochemistry

GC tissues were fixed with 10% formalin and incubated with specific primary antibodies, including EME1 (Bioss Bs-7861 R) and Ki-67 (Servicebio GB13030-2), following paraffin embedding. After incubating overnight at 4°C, the cells were incubated with HRP-linked secondary antibodies at ambient temperature. The sections were then stained with a 3,3-diaminobenzidine solution and hematoxylin prior to microscopy.

### Luciferase reporter assay

Cells were divided and cultured in 24-well plates for 1 d before plasmid transfection. On the day of transfection, the assays were performed according to the experimental design. The expression of the fluorescent marker gene (such as GFP) in cells was observed under a fluorescence microscope 24 h after transfection. The cells then underwent treatment with the ‘Dual-Luciferase® Reporter Assay System (E1910, Promega)’ kit, and the luciferase expression assay was performed.

### Immunofluorescence (IF)

An LSM 710 confocal microscope (Zeiss, Germany) was used to assess protein expression and localization. Immunofluorescence staining was performed on cells grown on glass coverslips. Cultured cells were fixed with 4% fresh paraformaldehyde for 30 min at 4°C and treated with 0.5% Triton X-100 in PBS (15 min). Sections were then blocked with 1% BSA for 1 h and incubated with primary antibodies overnight at 4°C. Next, the sections were treated with fluorescent-labeled secondary antibodies at a ratio of 1:200 in 1% normal donkey serum/PBS (1 h at ambient temperature, shielded from light). Images were acquired via laser scanning confocal microscopy (×40); these images represent three independent experiments.

### Kaplan–Meier analysis

Kaplan–Meier survival analysis was performed using KmPlot (http://kmplot.com/analysis) in 631 patients with GC. Kaplan–Meier curves were plotted for *EME1*. HRs and log-rank p-values were obtained for an OS assessment.

### Analysis of online datasets

Transcriptome sequencing data (FPKM) of GC (STAD) for a total of 407 cases were downloaded from The Cancer Genome Atlas (TCGA), including those of 375 cancer patients and 32 normal controls. Perl v5.26.1 and R v3.5.0 were used for the biochemical analysis. The R packages used mainly included edgeR, limma, ggpubr, ggplot2, survival, pROC, survminer, pheatmap, corrplot, circlize, clusterProfiler, org.Hs.eg.db, and enrichplot. The screening criteria for differential genes were logFoldChange = 1 and padj = 0.05. Screening criteria for co-expressed genes were |corFilter| = 0.4 and pFilter = 0.001.

### Statistical procedures

SPSS 22.0 (Chicago, IL) and GraphPad Prism 9.0 were used for data analyses. Data are expressed as the mean $\overset{ˉ}{x}$ ±SD of triplicate assays. The student’s t-test and analysis of variance (ANOVA) were used to compare two and multiple experimental groups, respectively. Chi-square or Fisher’s exact tests were performed for clinicopathological factors. Cox multiple regression analysis was used to identify parameters independently affecting survival and recurrence. Statistical significance was set at p < 0.05.

## Results

### EME1 *upregulation is common in GC*

**Figure 1.**
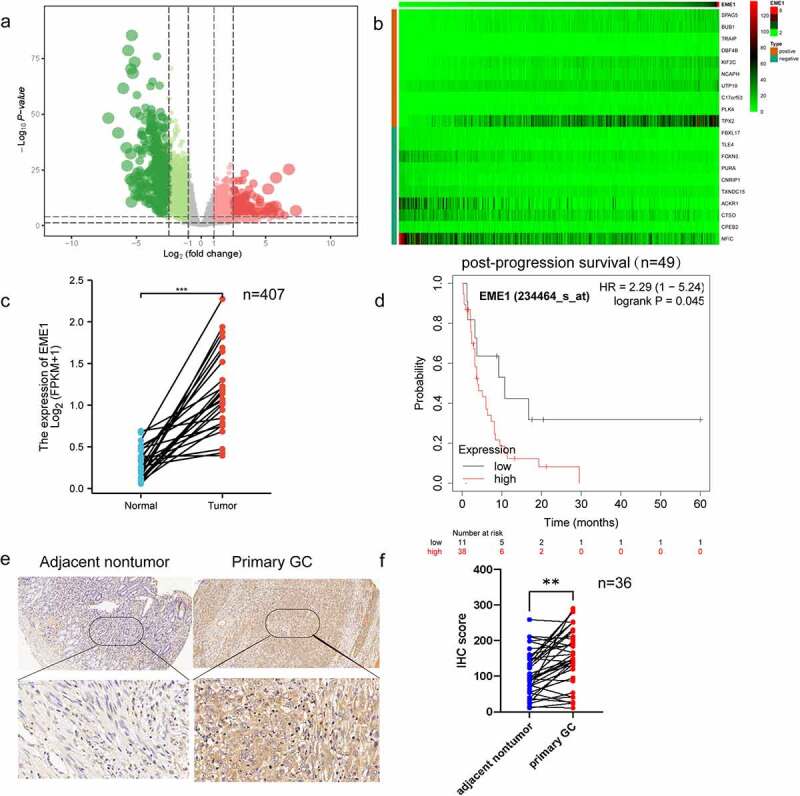
Upregulation of *EME1* reflects reduced survival in clinical GC. (a) Volcano plot was drawn to show differentially expressed genes, including 1110 upregulated and 1566 downregulated GC genes. (b) Top 10 upregulated and downregulated mRNAs between GC and adjacent tumor tissue samples in the heat map. (c) Quantitation of *EME1* amounts in 407 pairs of noncancerous and GC tissue specimens derived from the TCGA database. The y-axis reflects *EME1* staining intensity. (d) Kaplan–Meier curves for low and high *EME1* expression groups in GC cases. (e) Representative micrographs of the *EME1* expression in adjacent non-cancerous and primary GC tissue samples assessed via IHC staining. Nuclei appear blue and positive expression sites are brownish-yellow. Scale bars are 50 μm. (f) Semi-quantitative analysis of the *EME1* expression in adjacent noncancerous and primary GC tissue samples. ns: p > 0.05, *p < 0.05, **p < 0.01, ***p < 0.001. Data are χ ± SD from three measurements. All data points were measured in triplicate

The Cancer Genome Atlas (TCGA, http://www.cbioportal.org) was searched to obtain new mRNAs associated with gastric carcinogenesis. The analysis showed that 407 samples comprised 375 GC patients and 32 normal controls. The data were analyzed biometrically using Perl v5.26.1 and R v3.5.0 (2676 abnormally expressed mRNAs, including 1110 upregulated and 1566 downregulated mRNAs; the fold change was ≥2, p < 0.05), and a volcano plot was drawn (Figure 1(a)). Based on the screening criteria set for co-expressed genes, 266 co-expressed genes were screened, including 246 upregulated and 20 downregulated genes. The top 10 upregulated and downregulated co-expressed genes were selected separately for heat map generation, among which *EME1* was the most clearly regulated (Figure 1(b)) (ID:146956). Additionally, *EME1* was markedly upregulated in 407 primary GC tissue specimens than in paired adjacent noncancerous tissues (p < 0.001) (Figure 1(c)).

Prognostic value of *EME1* was assessed using the Kaplan–Meier survival analysis. In patients with poorly differentiated GC, post-progression survival (PPS) was markedly shorter in individuals with an elevated *EME1* expression than in those with a low *EME1* expression (Figure 1(d)). As lymph node metastasis (LNM) is an important prognostic predictor of GC outcomes and a major cause of GC deaths [13], we further investigated whether *EME1* expression was associated with lymph node metastasis in GC. As shown in Figure 1(e,f), EME1 was significantly upregulated in primary GC tissue specimens compared to matched normal tissue samples (p < 0.05). In addition, the comparative analysis of clinical prognostic parameters of 36 GC patients demonstrated that EME1 levels were positively correlated with poor prognostic parameters such as the differentiation degree and LNM (Table 1). In summary, the above data suggest that EME1 is upregulated in GC and may be associated with poor prognosis and LNM.

**Table 1.** Associations of EME1 expression in GC tissue samples with clinicopathological parameters

Variable Patient number (n = 36)		EME1 levels		P value	Chi-square
		Low (n = 10)	High (n = 26)		
Age (years)				1.0	0.043
≥60	20	5	14		
<60	16	5	12		
Gender				0.119	3.425
Female	13	6	7		
Male	23	4	19		
Tumor size				1.0	0.069
≥5 cm	12	3	9		
<5 cm	24	7	17		
Tumor invasion				0.140	1.527
T1	8	4	6		
T2	4	1	3		
T3	23	7	16		
T4a	1	0	1		
Lymph node metastasis				0.025	5.969
Presence	23	2	17		
Absence	13	8	9		
Differentiation grade				0.002	0.325
Well	10	2	3		
Moderate	6	2	2		
Poor	20	6	21		
Lauren classification				0.562	0.627
mixed	3	2	3		
diffuse	18	5	12		
intestinal	15	3	11		

### EME1 *silencing inhibits proliferation and induces apoptosis in cultured GC cells*

EME1 levels were markedly higher in all four GC cell lines than in human gastric mucosal cells; the highest expression was found in the MGC-803 and AGS cells (Supplementary Figure 1a). As demonstrated via qPCR, *EME1* mRNA levels were reduced in AGS and MGC-803 cells after transfection with siEME1 (p < 0.05), and knockdown efficiencies were 73% and 69%, respectively (Supplementary Figure 1b, c). *EME1* silencing was confirmed by immunoblot. Western blot was used to detect the knockdown of *EME1*. ImageJ was used to analyze the gray level of the WB images and the process was repeated thrice. The results showed that the gray level of the EME1 silencing group was significantly lower than that of the control group (the *EME1* expression in the silencing group was downregulated by 32%, compared to the control group). Tables and pictures are presented in Supplementary Excel 1.

**Figure 2.**
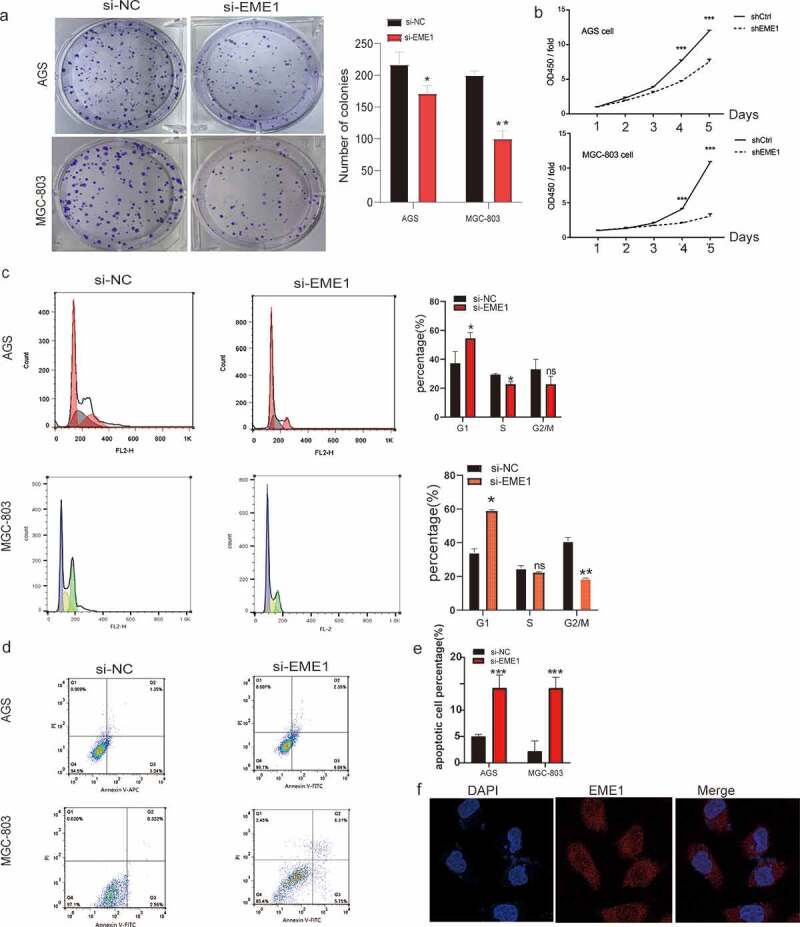
*EME1* silencing suppresses proliferation and enhances apoptosis in GC cells. (a, b) Cell proliferation as assessed by colony formation and CCK-8 assays, respectively. (c) Cell cycle distribution examined via flow cytometry. Cell rates in the G0/G1, S, and G2/M phases are shown. (d, e) AnnexinⅤ-FITC/PI double-staining and flow cytometry, respectively, showing increased apoptosis in *EME1*-silenced cells compared with null cells in both lines. (f) Immunofluorescence used to detect *EME1*; nuclei were stained with DAPI. Scale bars represent 10 µm. ns: p > 0.05; *p < 0.05; **p < 0.01; ***p < 0.001. Data are presented as the mean ±SD from three measurements

The colony formation assay showed that silencing *EME1* significantly reduced colony formation in MGC-803 and AGS GC cells (Figure 2(a)). The CCK-8 assay demonstrated that *EME1* knockdown inhibited the proliferation of AGS and MGC-803 cells (Figure 2(b)). In addition, flow cytometry demonstrated that *EME1* silencing promoted cell cycle arrest at the G1 phase in MGC-803 and AGS cells (Figure 2(c)). Furthermore, the knockdown of *EME1* induced apoptosis in the latter cell lines (Figure 2(d,e)). Using immunofluorescence, we confirmed that *EME1* was mainly localized in the cytoplasm, although it was also present in the nucleus (Figure 2(f)).

These findings suggest that *EME1* promotes tumorigenesis *in vitro* by inhibiting proliferation and promoting apoptosis.

### EME1 *promotes migration and invasion in GC cells* in vitro

**Figure 3.**
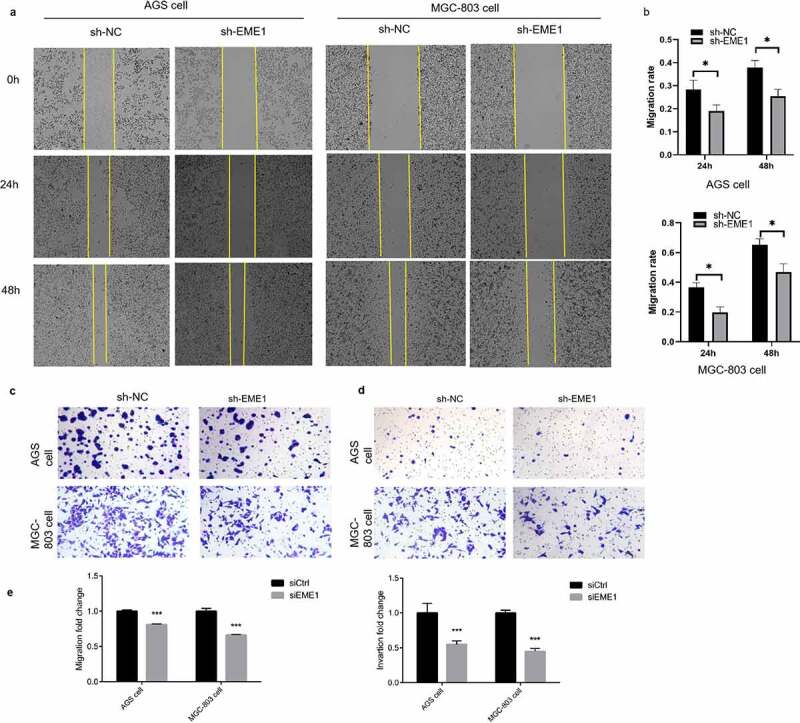
*EME1* promotes migration and invasion in cultured GC cells. (a, b) Scratch assay showing reduced migration rates in the AGS and MGC-803 cell lines, respectively, compared with the NC group 24 h after downregulation of *EME1*. (c, e) Transwell assay demonstrating that *EME1* silencing remarkably decreased the migration rates of the AGS and MGC-803 cell lines, respectively, compared with the control. (d, e) Transwell assay showing decreased cell invasion and migration, respectively following the downregulation of *EME1*. ns: p > 0.05; *p < 0.05; **p < 0.01; ***p < 0.001. Data are presented as the mean ±SD from three measurements

The scratch assay demonstrated that *EME1* silencing decreased the migratory ability of MGC-803 and AGS cells (Figure 3(a,b)). The Transwell assay consistently showed that *EME1* knockdown markedly suppressed cell migration and invasion (Figure 3(c-e)).

### EME1 *promotes tumor cell proliferation* in vivo

**Figure 4.**
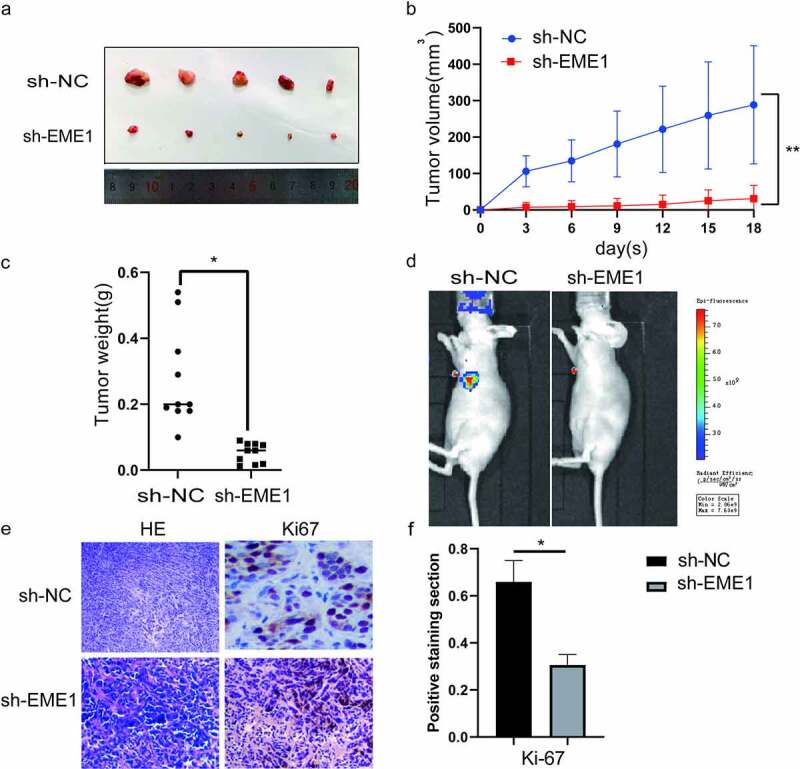
*EME1* silencing inhibits tumor cell proliferation *in vivo.* (a) AGS xenograft sizes at 30 d after treatment with Si-*EME1* and NC transfected cells. (b) Tumor growth curves of AGS xenografts in the Si-*EME1* and NC groups. (c) Tumor weights in the Si-*EME1* and NC groups. (d) Representative bioluminescence images in both groups eight weeks following the tail vein injections of cells. (e) Immunohistochemical staining used to detect Ki-67 levels in transplanted xerographs of the Si-EME1 and NC groups (original magnification, ×200). (f) Mean optical density values of Ki-67 in both groups. Data are χ ± SD. t-test: *P < 0.05, **P < 0.01, ***P < 0.001. All data points were measured in triplicate

To investigate the function of *EME1 in vivo*, stably transfected AGS cells were inoculated into the lateral abdominal subcutis of nude mice. Thirty days later, tumor volumes were remarkably reduced in sh-EME1-transfected AGS cells compared with the NC group (Figure 4(a)). Accordingly, tumors showed a time-dependent growth rate reduction in the sh-EME1 group (Figure 4(b)). Tumor weights on the last day of study were clearly reduced after transfection with sh-EME1, compared to that in the NC group (Figure 4(c)). The small animal live imaging system (Lumina III, Perkin Elmer) demonstrated that the luciferase flux count was significantly lower in the sh-EME1 group than that in the sh-NC group (p < 0.05) (Figure 4(d)). Additionally, the effect of *EME1* on Ki-67 expression in transplanted tumor tissues was examined via immunohistochemistry. As shown in Figure 4(e,f), *EME1* silencing overtly downregulated the Ki-67 expression. In conclusion, these results suggest that silencing *EME1* significantly suppresses gastric carcinogenesis *in vitro* and *in vivo*.

### EME1 *regulates Akt/GSK3B/CCND1 signaling*

**Figure 5.**
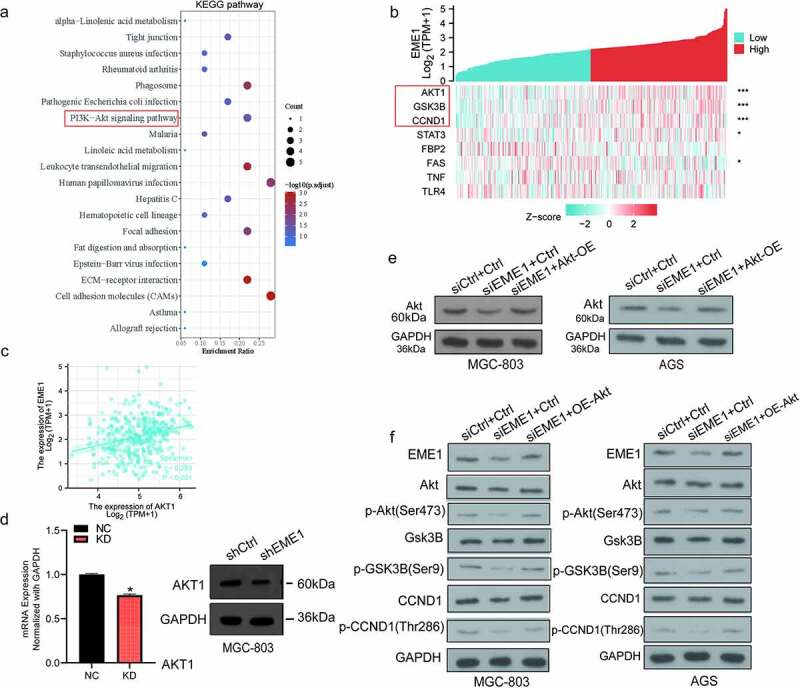
*EME1* regulates Akt-GSK3B-CCND1 pathway. (a) KEGG enrichment analysis of 20 important pathways regulated by *EME1* in GC cells. (b) Heat map of *EME1* co-expression in GC tissues. (c) *EME1* and Akt1 as positively correlated in GC tissues (P < 0.001). (d) qRT-PCR and immunoblot analyses of *EME1* and Akt1. (e) Immunoblot of Akt in the rescue experiment. (f) EME1 knockdown downregulating major Akt pathway effectors. *P < 0.05, **P < 0.01

To investigate the major downstream pathways modulated by *EME1* and contributing to GC carcinogenesis, an analysis of the Kyoto Encyclopedia of Genes and Genomes (KEGG; http://www.genome.jp/kegg) was performed. The results revealed that downregulated genes may be associated with various pathways, with PI3K/Akt signaling as the top enriched pathway (Figure 5(a)). RNAseq data in the level 3 HTSeq-FPKM format in the TCGA STAD (gastric cancer) project were used for *EME1* co-expression analysis. The results revealed that the expression of *EME1* was positively correlated with Akt1, GSK3B, and CCND1 (p < 0.001), but not with P13 K in GC tissues. These findings further corroborate our hypothesis that *EME1* affects the essential genes of the Akt pathway (Figure 5(b)). In addition, the *EME1* expression was found to be correlated with Akt1 in GC tissues (p < 0.001) (Figure 5(c)).

The expression of Akt in MGC-803 cells was examined using qRT-PCR. The lentivirus-transfected KD group had lower levels than the lentivirus-transfected NC group. An Akt overexpression plasmid was constructed, and immunoblotting demonstrated that *EME1* expression was decreased following *EME1* knockdown (Figure 5(d)). However, the expression level of *EME1* was restored to a certain extent with *EME1* knockdown and Akt overexpression performed simultaneously (Figure 5(e)). Subsequently, Silencing *EME1* in two gastric cancer cell lines decreased the expression of Akt, CCND1, and GSK3B as well as the phosphorylation levels of Akt, CCND1, and GSK3B. However, when *EME1* knockdown and Akt overexpression occurred at the same time, the expression and phosphorylation levels of Akt, CCND1, and GSK3B were restored to a certain extent. Therefore, we confirm that the *EME1* knockdown downregulated the major Akt pathway effectors, indicating that *EME1* silencing suppressed the latter pathway (Figure 5(f)). These findings suggest that *EME1* regulates the Akt/GSK3B/CCND1 signaling pathway.

### EME1 *silencing inhibits GC progression* in vitro *by downregulating Akt*

**Figure 6.**
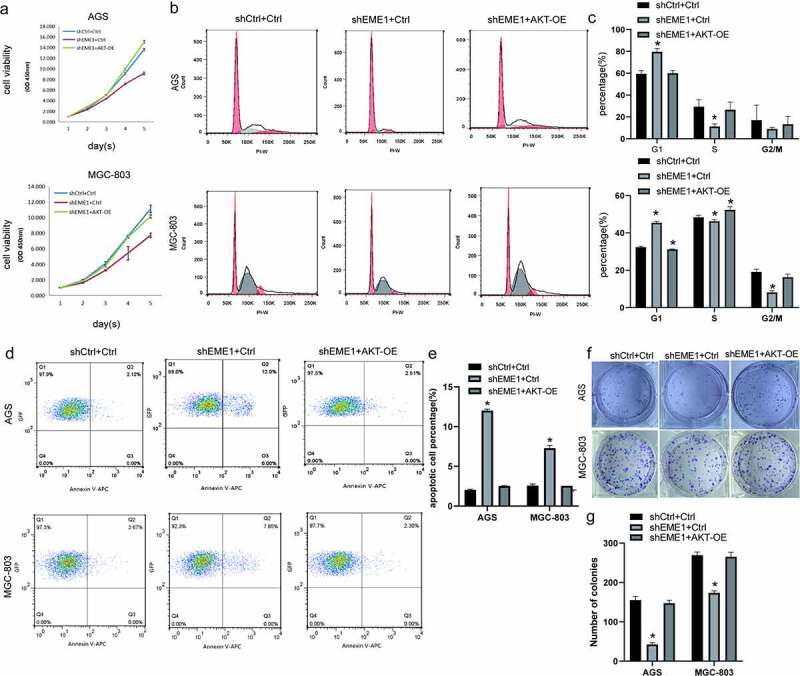
Silencing of *EME1* inhibits GC progression *in vivo* through downregulation of Akt. (a) Overexpression of Akt promoting the proliferation of AGS and MGC-803 cells, in contrast to the *EME1* silencing group. (b, c) G0/G1 and S and G2/M phase rates, respectively, following Akt overexpression; these rates show opposite trends to the results of the *EME1* silencing group, as shown via flow-cytometry. (d, e) Flow cytometry demonstrating that the overexpression of Akt affected apoptosis in AGS and MGC cells, respectively, with a trend opposite to that of the *EME1* silencing group. (f, g) Colony formation assay. The overexpression of Akt altered the number of visible colonies in AGS and MGC cells, respectively with a trend opposite to that of the *EME1* silencing group. All assays were repeated thrice. Data are presented as the mean ±SD. *p < 0.05

Rescue experiments were conducted to examine the impact of *EME1*-mediated Akt on GC progression in cultured cells. *EME1-*overexpressed Akt was silenced in AGS and MGC-803 cells. CCK-8, clonogenic, and flow cytometry assays showed increased cell proliferation (Figure 6(a)), reduced G1 and elevated S phase cell rates (Figure 6(b,c)), decreased apoptosis (p < 0.05) (Figure 6(d,e)), and increased clone numbers (Figure 6(f,g)) with *EME1* knockdown and Akt overexpression performed simultaneously; this was in contrast to the knockdown results in the *EME1* group. These results suggest that *EME1* promotes GC development by positively regulating Akt in GC cells.

### EME1 *is a direct target of MYB*

**Figure 7.**
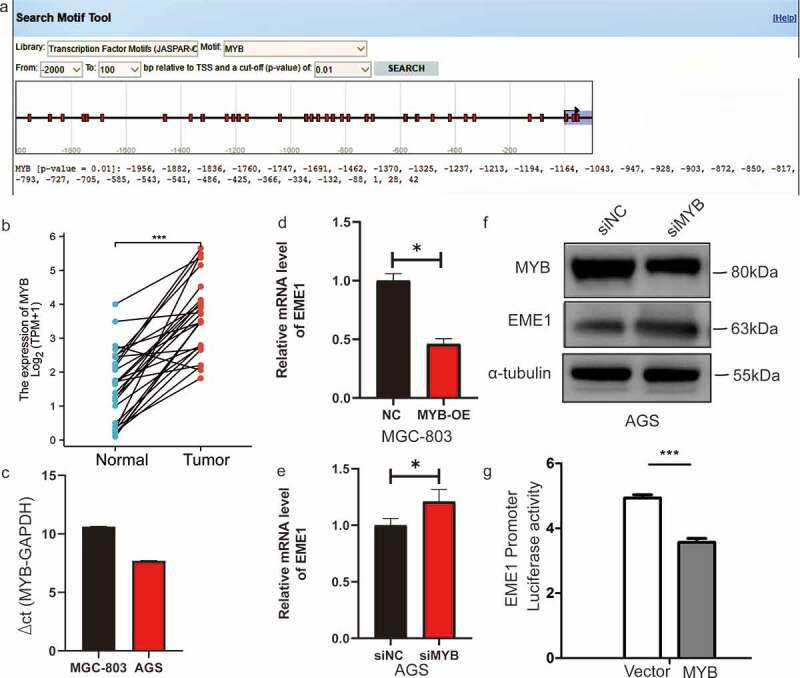
MYB interacts with the *EME1* promoter and negatively regulates *EME1* in GC cells. (a) Predicted binding sites of *EME1* to MYB. (b) Quantification of MYB levels in 407 pairs of noncancerous and GC tissue specimens derived from the TCGA database. The y-axis shows the MYB staining intensity. (c) *MYB* mRNA expression levels in GC cells (MGC-803 and AGS), as detected by qRT-PCR. (d, e) Results of overexpression (d) or knockdown MYB (e) showing that MYB negatively regulated EME1 expression. (f) EME1 and MYB as examined by Western blotting in AGS cells transfected with si-Ctrl and si-MYB. (g) MYB transfected into 293 T cells, and the promoter activity of *EME1* was assessed using a dual-luciferase reporter gene assay. *P < 0.05, **P < 0.01, ***P < 0.001

To further explore the possible mechanism by which *EME1* contributes to GC, the potential transcription factors of *EME1* were examined using the online database JASPAR (http://jaspar.genereg.net) [14]. MYB (ID:4602) contained the binding sequence of the *EME1* promoter (Figure 7(a)). The MYB binding site sequence identification is shown in Supplementary Figure 2c. The length of the *EME1* promoter was 2012bp, and the cloning vector was pGL4.10. The promoter sequence was synthesized by the whole gene and cloned into the pGL4.10 vector via the NheI and XhoI digestion sites. The insertion sequence of the transcription factor was listed as a complete coding sequence (CDS) region, which was synthesized by the whole gene and cloned into the PEGFP-N1 vector via the XhoI and BamHI digestion sites. The *EME1* startup subsequence is detailed in Supplementary Table 3. *MYB* is expressed in GC metastatic foci [15]. However, the interaction between *MYB* and *EME1* in GC is unclear.

First, *MYB* was clearly upregulated in 407 primary GC tissue specimens versus paired adjacent noncancerous tissue (p < 0.001) (Figure 7(b)). *MYB* expression levels were elevated in MGC-803 and AGS cells (Figure 7(c)). qPCR demonstrated that *EME1* was markedly downregulated (p < 0.001) after an overexpression of *MYB* in MGC-803 cells (Figure 7(d)), and the mRNA and protein expression levels of *EME1* increased following interference with MYB in AGS cells (Figure 7(e,f)), thereby indicating that MYB regulated *EME1*. A dual-luciferase reporter gene assay showed that MYB significantly inhibited *EME1* promoter activity (Figure 7(g)). These findings suggest that MYB directly targets and negatively regulates *EME1* in GC cells.

## Discussion

In this study, bioinformatics analysis results showed that *EME1* was highly expressed in gastric cancer tissues and was associated with poor prognosis of gastric cancer. Further validation results revealed that the expression of *EME1* increased both in gastric cancer cell lines, including AGS and MGC-803, as well as gastric cancer tissue, and that *EME1* promoted the proliferation and metastasis of gastric cancer and inhibited apoptosis both *in vitro* and *in vivo*. These changes may be caused by the MYB/EME1/Akt pathway. EME1 is a heterodimeric endonuclease that contributes to DNA repair and is associated with drug resistance [16,17]. However, its role in GC has rarely been reported as an important prognostic marker [18]. In this study, we revealed that *EME1* was highly expressed in human GC tissue specimens as well as GC cells, which is indicative of a key function for *EME1* overexpression in GC tumorigenesis. Importantly, elevated EME1 levels were associated with reduced GC patient survival, thereby suggesting that *EME1* overexpression is a biomarker of poor prognosis in GC. The expression analysis of *EME1* in GC cases suggested that high EME1 levels in GC may promote tumor formation by enhancing cell proliferation and metastasis. Furthermore, we demonstrated that Akt signaling, a key pathway primarily involved in cell proliferation and invasion, was suppressed upon *EME1* knockdown [19,20]. This result is consistent with our previous hypothesis suggesting that Akt signaling may be downstream of *EME1*. Therefore, we hypothesized that *EME1* may regulate proliferation and metastasis in GC cells via this pathway. EME1 also plays a critical role in cultured cells, but its role in GC is unclear. Clinicopathological characterization revealed that *EME1* may induce proliferation and metastasis in GC. Following the knockdown of *EME1*, the major biological functions of Akt were inhibited *in vitro*, and Akt overexpression reversed the inhibitory effects of *EME1* on cell proliferation, further confirming that Akt is a molecular target of *EME1*. These results indicate that the Akt expression and function are controlled by *EME1 in vitro*.

Upon further exploring the mechanism of *EME1* overexpression in GC, *EME1* was found to be upregulated in GC cells, thus indicating that it might be transcriptionally regulated for specific overexpression in GC. Because this regulation frequently occurs in the *EME1* promoter, we hypothesized that certain tumor-specific cytokines may specifically bind to the *EME1* promoter and thus upregulate *EME1*. Using the JASPAR database, we predicted that MYB may be involved in this process. MYB is considered a prognostic biomarker of *Helicobacter pylori*-positive GC, but this has not been confirmed experimentally [21]. As transcription factors, MYB family members, that is, A-MYB, B-MYB, and C-MYB, are highly expressed in multiple malignancies such as non-small cell lung cancer, colon cancer, and hepatocellular carcinoma [22–24], with key roles in cell proliferation [25], cell cycle regulation [26], apoptosis, and tumor progression [27]. However, the expression of MYB in GC remains unclear. To further verify whether MYB binds to the *EME1* promoter, a luciferase activity assay was carried out in 293 T cells. The results showed that MYB was bound to the *EME1* promoter, and enhanced binding was detected in GC cell lines. Enhanced binding may therefore contribute to *EME1* overexpression. We demonstrated that MYB inhibited *EME1* promoter activity. Furthermore, *EME1* RNA levels were suppressed following the overexpression of MYB, whereas *EME1* knockdown increased *MYB* RNA levels, thereby suggesting that *MYB* regulates *EME1* at the transcriptional level. These results suggest that *EME1* expression in GC tissues decreases with increasing *MYB* levels in a negative feedback mechanism. Therefore, targeting *EME1* may be used to treat GC. However, a limitation of this study was the lack of human tissues or transcriptome level studies as a whole.

The biological function of *EME1* was assessed using a mouse xenograft model. Consistent with the *in vitro* experiments described above, *EME1* promoted the proliferation and metastasis of GC cells *in vivo*. In conclusion, *EME1* overexpression is a marker of poor prognosis of GC and controls cell proliferation and metastasis through Akt/GSK3B/CCND1 signaling. In GC, MYB interacts with the *EME1* promoter, thereby controlling *EME*1 expression transcriptionally.

## Conclusions

Overall, *EME1* is a regulator of oncogenic DNA damage and repair, with elevated levels correlating with poor GC prognosis. *EME1* exerts pro-tumorigenic effects by activating the Akt/GSK3B/CCND1 pathway to increase proliferative, migratory, and invasive abilities in GC cells, while inhibiting apoptosis. Our research suggests that *EME1* may be an important molecular marker of gastric carcinogenesis and could represent a novel candidate gene for the prognosis and treatment of GC. Therapeutically, MYB mediated *EME1* activation promoted tumorigenesis in a GC cell line, orthotopic xenograft gastric cancer model. Targeting *EME1*/Akt provides a therapeutic strategy for GC.

## Supplementary Material

Supplemental Material

## Research highlights

•*EME1* was highly expressed in GC tissues compared to adjacent non-tumor tissues.
•*EME1* promoted GC cell proliferation, migration, and invasion.
•MYB negatively regulates *EME1* in GC cells via the Akt/GSK3B/CCND1 pathway.
•Overexpression of *EME1* correlates with poor prognosis of GC and LNM.
