# Supplementary material for: Essential meiotic structure-specific endonuclease1 (EME1) promotes malignant features in gastric cancer cells via the Akt/GSK3B/CCND1 pathway
Source: Bioengineered. 2021 Dec 11;12(2):9869–84. doi: 10.1080/21655979.2021.1999371 (PMC8810030; doi:10.1080/21655979.2021.1999371)
Supplement: Supplemental Material [file KBIE_A_1999371_SM9616.zip › supplementary/Supplementary Figure legend.docx]

**Supplementary Figure S1. *EME1* background expression and knockdown efficiency**

(a.) *EME1* mRNA levels in different samples as detected by real-time quantitative PCR. (b.) *EME1* mRNA levels as assessed by qRT-PCR following transfection of the MGC-803 and AGS cells with overexpression vectors and siRNAs. (c.) Changes in EME1 protein levels following interference, as assessed by immunoblotting. ns, p>0.05; *p<0.05; **p<0.01; ***p<0.001. Data are presented as the mean ±SD from three measurements.

**Supplementary Figure S2.** Plasmid transfection

Fluorescence microscopy used to detect the transfection efficiency in the PEGFP-N1 plasmid single transfer group. Twenty-four hours after transfection with 1 ug plasmid, the transfection efficiency was greater than 80%. (a.) Before transfection. (b.) Experimental group after transfection. (c.) MYB sequence logo. All data points were measured in triplicate.

**Supplementary Figure S3. Animal model study**

(a.) Image of cells before inoculation, with a microscope magnification 200X. (b.) Experimental animal models. All data points were measured in triplicate.
