# Supplementary material for: Essential meiotic structure-specific endonuclease1 (EME1) promotes malignant features in gastric cancer cells via the Akt/GSK3B/CCND1 pathway
Source: Bioengineered. 2021 Dec 11;12(2):9869–84. doi: 10.1080/21655979.2021.1999371 (PMC8810030; doi:10.1080/21655979.2021.1999371)
Supplement: Supplemental Material [file KBIE_A_1999371_SM9616.zip › supplementary/Supplementary table_3.docx]

**Supplementary Table 3: The EME1 startup subsequence**

| **The EME1 startup subsequence** |
| --- |
| GCTAGCATTACAGGCCTCACCCATGACTTTCTCAGCTGTCCACTCATCCACTGCTCACCAAATACTAGTAAACCAAAAGGATGGCTGGCCTAAAAGTGGCTCCCACAGGGTCCTGGCCATTTCAGAAGGCTGGGCCCTTTCTTCTCAAAGCACATCAAGCAGAGGTCCAACCATGACCCACACACTGCCCTTGTTAATCTGACCTCTCCATACTCATTCCAAAGTGCTCAATAGCATTCATTTGCAGAAGTAAAATGAACTAAAATTCATACTAAGATGGTAGTCCCTTGCTGTATGACAAGCCTAGGGGTGCAACTCCTTGCCTGCAGGTCTCCAGACACAGCCATGATTTAAGGTCCATGCCAGTGTCTTTAGAGAAGCCTTGTGGGTCTCTGGAGCCTATAAGTTCAGAGTTCCTGAAGCCTGGGTTTCTGACATTTCCATGACACCAGAGACTAGCTGGGGCAAAGGACCAGAGATAAGGACTCAGAAAGACAACATGTTAAAACATGCCCGGCGCCAGGATCTACCTACCTTTAAACATCTATTCATTCATTTACATACTTACTAAGCTCAGGAGCTGGGCTTTTACTTTACTTACATTATCTCACTCACAAATCTTTGCTCTTCACCCTACTCCCCATCCTCCTCCTCAAAGTAAATGGTACCACCATTCATCCAGTTGCTTAGGCAGAAACCAGAGTCATTGCAGATTCCTTCTCTTTGCTGTCCTCACATATTGTTCACCAGCAAGCTCTGTGGATGCTAACTCCAAAACAGATCTCAAATTCATCCGTTTTCCTCCAAGTTCACTGCCACCACAAACATCTTGGCTAAATCATCTCTCCTCAGGGCTATTACGACTTTCCATTTGATTTCCTCATTTCCACTCCTATCCCCTTTAAATTTACATAAGAGCAATAGTTTGCTTCTCAAAATGTAAAACTGGCTCAAGTCACTCTCCTGTCCCTCACTCTACACAGGTCTAGGTCTCCTGTCATTCTTTTTTTTTTGGGGGGGGGGGATAGGGCCCCATTCTGTCACCCAGGCTGGAGTGCAGTGGTATGAACATAGCTCACTGCAACCTCGACCTCCCGAGCTCAGGTGATCCTCCCACCTCAGCCTCCTGAGTATCTGAGACCACAGGCAAGCGCCACTACACCCAGCTAATTTTTGTATTTTTTGTAGAGATGGGGTTTTGCCATGTTGCCCAGTCGGTCTTGAACTCCTGGGCTCAAGCGATCCGCCCACCTAGGCCTCCCAAAGTGTTGGGACTACAGGCGTGAGCCATCACGCCCCGCCTGTCATTCTTTAGGTTTTAAATGTTATCTCCTCAGAAGCCTTCCTTCACCGTCCAATCTAAAAAAAGACACACACACAGACACACATTATCCTTCTTCACAGCACTTGCCAGTCTACATCTACTTTATATGTTCATTTATTGCCTTATGCACCAGAGAGAACCGTTCGGGGGCTAGAAGCCAAGTCTTCACGTTTTCTGCTGATTTTCCGAACACCTACCGCCGTGTCTAGCACAAAGTAAGACCTCAATCTGCTGAATCAATAGATTAATTTTTTCAACCCCAATATGCCAATGTTACTAATACTTCTACAGAGAAAGGACCCACCCAAATCCCCAACCGCCAAGGAACCCGTCCGTGCTGCTGAGGCAGGAGAAAGACAAGTGCTCTTCAAACGCCCTCACTCACGGCTCCACCCAGGATGTGTCACGTGACCAGCGACACCGTCCCTCAAACCTGGAGTACCCCACACACTTCCCCTCGCATCCAAGGTCCTCCGCGGGGATCAGTGTCACCTCCTTCCCTCCCTGCTGGAGCTCCACTCTGCCTCCCCGCCTCACCCCGCTCTGACTACTGCGTCCCATTACCGGCTGTCCGGGTCCTCAGCGCCAACACCACCGACGCCATGCTTTCGATCACTCACTTCCGGTCTCGAAAAGTTTCTACTTCCGCTATCAGGAGATCTACTTCCGGGCCCTGCGTGGCCTCGAG |
