# Supplementary figures and images for: Essential meiotic structure-specific endonuclease1 (EME1) promotes malignant features in gastric cancer cells via the Akt/GSK3B/CCND1 pathway
Source: Bioengineered. 2021 Dec 11;12(2):9869–84. doi: 10.1080/21655979.2021.1999371 (PMC8810030; doi:10.1080/21655979.2021.1999371)

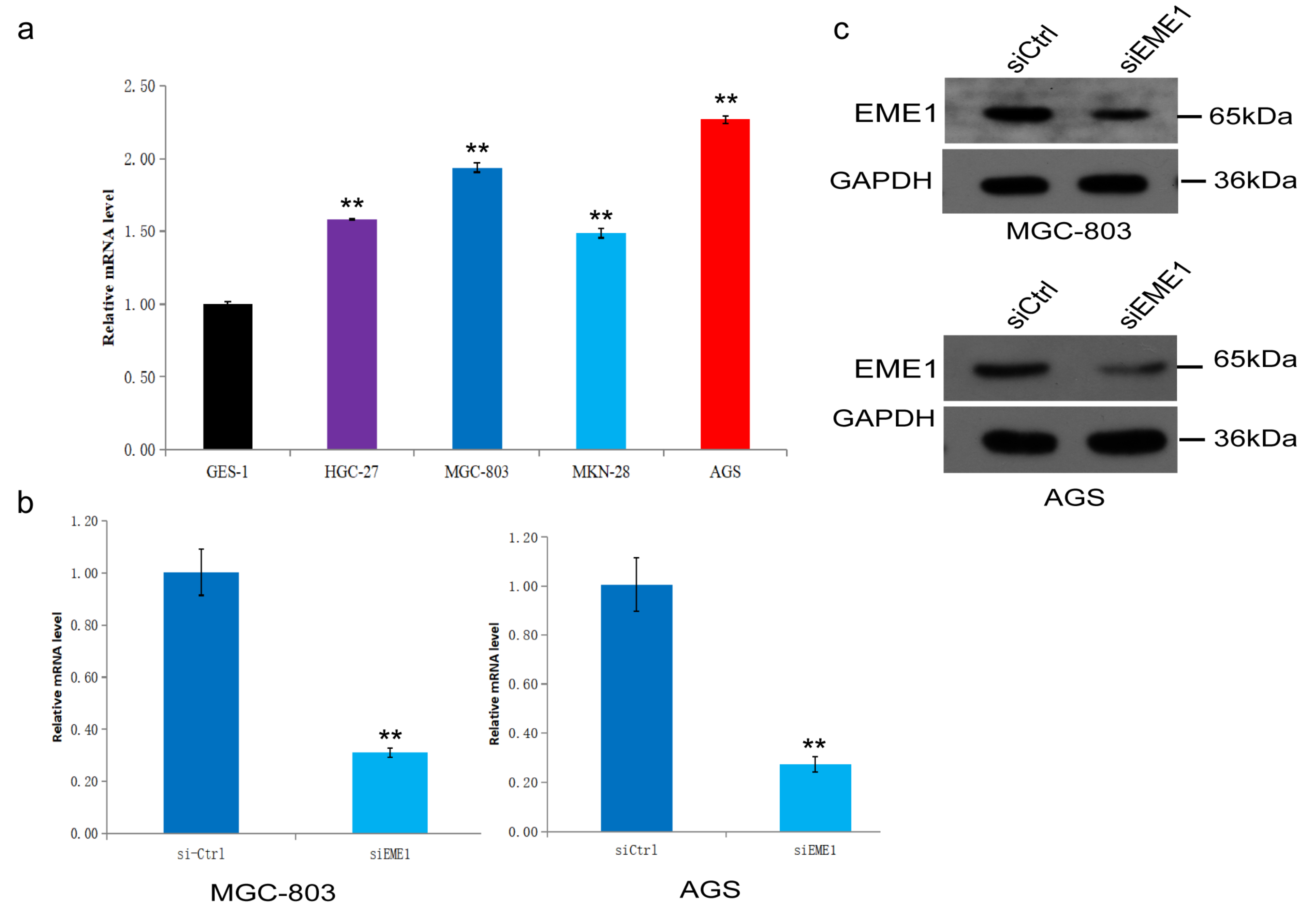

Supplement: Supplemental Material [file KBIE_A_1999371_SM9616.zip › supplementary/Fig_S1.jpg]

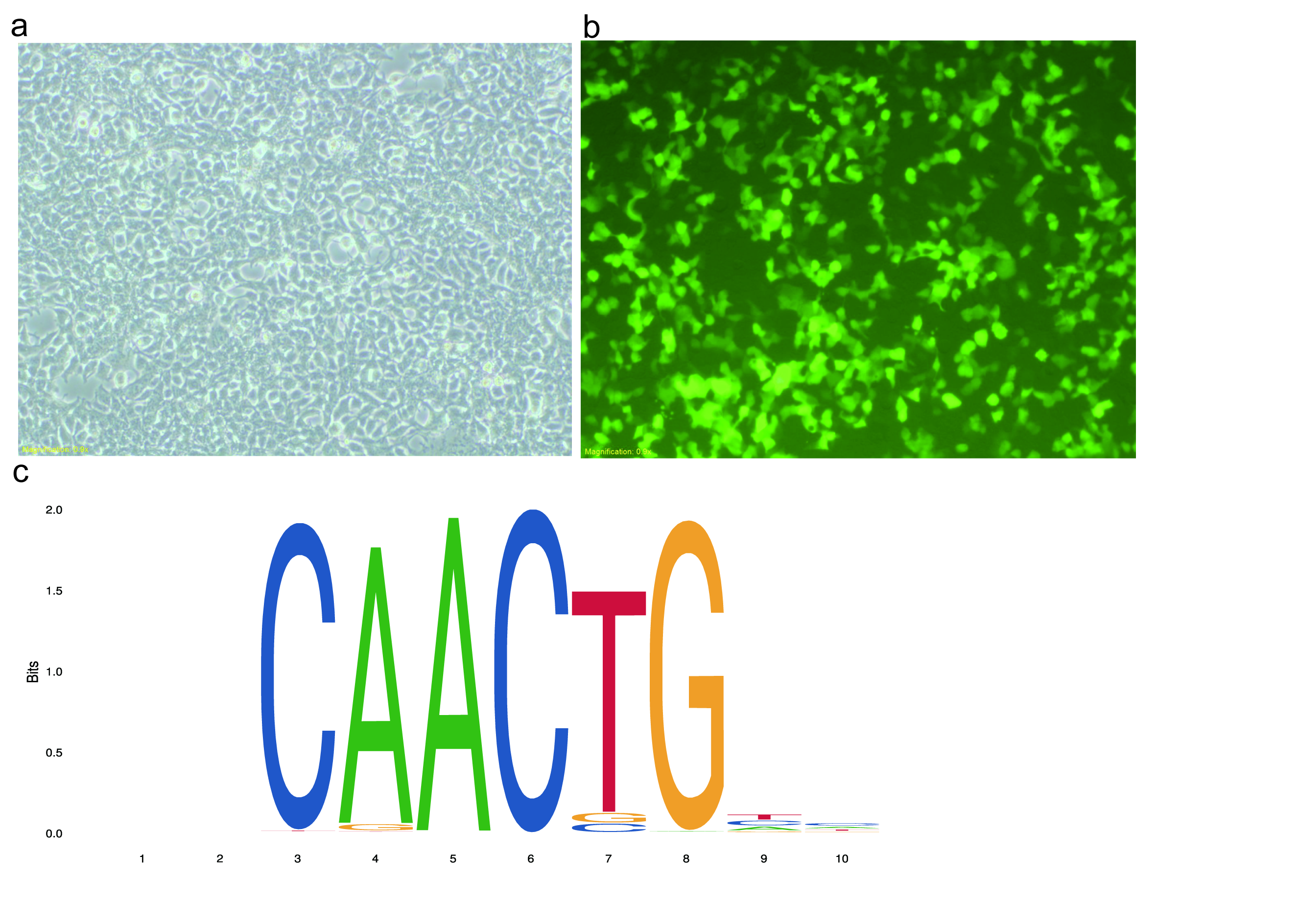

Supplement: Supplemental Material [file KBIE_A_1999371_SM9616.zip › supplementary/Supplementary Figure_2.tif]
