# Supplementary material for: Essential meiotic structure-specific endonuclease1 (EME1) promotes malignant features in gastric cancer cells via the Akt/GSK3B/CCND1 pathway
Source: Bioengineered. 2021 Dec 11;12(2):9869–84. doi: 10.1080/21655979.2021.1999371 (PMC8810030; doi:10.1080/21655979.2021.1999371)
Supplement: Supplemental Material [file KBIE_A_1999371_SM9616.zip › supplementary/Supplementary table_1.doc]

**Supplementary Table 1: The sequences of siRNAs and shRNAs.**

| **SiRNA Targets** | **Sequences** |
| --- | --- |
| EME1 siRNA-1 sense | 5′-CCUGGACAGCACUAUGAAATT-3 |
| EME1 siRNA-1 antisense | 5′- UUUCAUAGUGCUGUCCAGGCT-3′ |
| EME1 siRNA-2 sense | 5′- GGAAAUGGCCAGUGCAGUUTT-3′ |
| EME1 siRNA-2 antisense | 5′- AACUGCACUGGCCAUUUCCAG-3′ |
| EME1 siRNA-3 sense | 5′- GCCACAUCUCUCUUUAGAUTT-3′ |
| EME1 siRNA-3 antisense | 5′- AUCUAAAGAGAGAUGUGGCTG-3′ |
| MYB siRNA-1 sense | 5′- GAUAGAGCUUGUACAGAAATT-3′ |
| MYB siRNA-1 antisense | 5′- UUUCUGUACAAGCUCUAUCTT-3′ |
| MYB siRNA-2 sense | 5′- GAAAUACGGUCCGAAACGUTT-3′ |
| MYB siRNA-2 antisense | 5′- ACGUUUCGGACCGUAUUUCTT-3′ |
| MYB siRNA-3 sense | 5′- UGCUAUCAAGAACCACUGGTT-3′ |
| MYB siRNA-3 antisense | 5′- CCAGUGGUUCUUGAUAGCATT-3′ |
| NC-siRNA sense | 5′-UUCUCCGAACGUGUCACGUdTdT-3′ |
| NC-siRNA-3 antisense | 5′-ACGUGACACGUUCGGAGAAdTdT-3′ |
| shEME1 | 5'-GATCCGGAAATGGCCAGTGCAGTTCTTCCTGT  CAGAAACTGCACTGGCCATTTCCTTTTTG-3' |
| shRNA NC | 5'-TTCTCCGAACGTGTCACGT-3' |
