# Supplementary material for: Essential meiotic structure-specific endonuclease1 (EME1) promotes malignant features in gastric cancer cells via the Akt/GSK3B/CCND1 pathway
Source: Bioengineered. 2021 Dec 11;12(2):9869–84. doi: 10.1080/21655979.2021.1999371 (PMC8810030; doi:10.1080/21655979.2021.1999371)
Supplement: Supplemental Material [file KBIE_A_1999371_SM9616.zip › supplementary/Supplementary table_2.doc]

**Supplementary Table 2: The sequences of PCR primers.**

| **Primers** | **Sequences** |
| --- | --- |
| EME1 | Forward: 5′-CTCCATGATACCCCAGAGAGG-3′ |
|  | Reverse: 5′-CCTGGACCTTCTGACTCGG-3′ |
| MYB | Forward: 5′-GAAAGCGTCACTTGGGGAAAA-3′ |
|  | Forward: 5′-TGTTCGATTCGGGAGATAATTGG-3′ |
| AKT1 | Forward: 5′-TCCTCCTCAAGAATGATGGCA-3′ |
|  | Reverse: 5′-GTGCGTTCGATGACAGTGGT-3′ |
| CCND1 | Forward: 5′-TGGAGCCCGTGAAAAAGAGC-3′ |
|  | Reverse: 5′-TCTCCTTCATCTTAGAGGCCAC-3′ |
| GAPDH | Forward: 5′-TGACTTCAACAGCGACACCCA-3′ |
|  | Reverse: 5′-CACCCTGTTGCTGTAGCCAAA-3′ |
| shEME1 | Forward:5'-GATCCGGAAATGGCCAGTGCAGTTCTTCCTGTCAG  AAACTGCACTGGCCATTTCCTTTTTG-3' |
|  | Reverse: 5'-AATTCAAAAAGGAAATGGCCAGTGCAGTTTCTGA  CAGGAAGAACTGCACTGGCCATTTCCG-3' |
